# Examining Infant and Child Neurodevelopmental Outcomes After Lyme Disease During Pregnancy

**DOI:** 10.3390/pathogens13121029

**Published:** 2024-11-22

**Authors:** Meagan E. Williams, David A. Schwartz, Roberta L. DeBiasi, Sarah B. Mulkey

**Affiliations:** 1 Zickler Family Prenatal Pediatrics Institute, Children’s National Hospital, Washington, DC 20010, USA; sbmulkey@childrensnational.org; 2 Division of Pediatric Infectious Diseases, Children’s National Hospital, Washington, DC 20010, USA; rdebiasi@childrensnational.org; 3 Perinatal Pathology Consulting, Atlanta, GA 30342, USA; davidalanschwartz@gmail.com; 4 Department of Pediatrics, The George Washington University School of Medicine and Health Sciences, Washington, DC 20037, USA; 5 Department of Neurology, The George Washington University School of Medicine and Health Sciences, Washington, DC 20037, USA

**Keywords:** tick-borne disease, congenital infection, child development, post-treatment Lyme disease syndrome, PTLDS, chronic Lyme disease, *Borrelia burgdorferi*, placental pathology

## Abstract

Lyme disease is the most common vector-borne disease in the United States. Recent environmental and socioecological changes have led to an increased incidence of Lyme and other tick-borne diseases, which enhances the urgency of identifying and mitigating adverse outcomes of Lyme disease exposure. Lyme disease during pregnancy, especially when untreated, may lead to adverse pregnancy and neonatal outcomes; however, long-term child outcomes following utero exposure to Lyme disease have not yet been systematically assessed. This concise review describes the current state of knowledge of Lyme disease as a congenital infection and the potential effects of in utero exposure to Lyme disease infection on the neurodevelopment of infants and children. We highlight the importance of distinguishing between acute Lyme disease and a chronic condition termed Post-Treatment Lyme Disease Syndrome, as the impacts of both conditions on the developing fetus and subsequent child development may differ. The importance of placental pathology for patients with acute or chronic symptoms of Lyme disease in pregnancy is explored. Future research aiming to understand and protect neurodevelopment after antenatal Lyme disease must carefully collect potentially confounding variables such as symptomatology and treatment, use clear and standard case definitions, and follow children into school-age and beyond.

## 1. Introduction

The annual number of tick-borne illnesses has more than doubled during the previous two decades as a result of increasing tick populations and environmental changes such as climate change and altered land use patterns [1,2]. Lyme disease, caused by the spirochete *Borrelia burgdorferi* sensu lato, is the most common tick-borne disease in the United States. Recent surveillance data indicate that over 62,000 cases were formally reported to the Centers for Disease Control and Prevention (CDC) in 2022; however, like other public health surveillance data, this measure likely underestimates the true incidence of Lyme disease in the United States, which has recently been estimated by other measures at over 475,000 new diagnoses per year [3,4]. Commercial insurance claims data from 2010 to 2018 suggest an annual incidence of Lyme disease diagnosis ranging between 49 and 88 per 100,000 enrollees with a median of 73 per 100,000, which is 62% higher than the median incidence of 45 per 100,000 reported between 2005 and 2010 [5]. This trend is expected to continue, particularly in regions that border areas with high endemicity [5].

With increasing incidence, prevalence, and awareness of Lyme disease, it has become increasingly important to prevent, identify, and address short- and long-term outcomes of Lyme disease infection, including potential congenital transmission of *B. burgdorferi* to developing fetuses. Lyme disease can also result in a chronic condition termed Post-Treatment Lyme Disease Syndrome (PTLDS) in a subset of patients, in which patients have prolonged symptoms such as fatigue, body pain, and cognitive difficulties for at least six months after initial treatment of their infection [6]. Investigation of both acute Lyme disease and PTLDS as potential health hazards during pregnancy has been identified as a priority research topic [7,8]. Once perinatal and childhood neurodevelopmental outcomes are identified, appropriate follow-up and treatment guidelines can be developed to help better support the needs of families impacted by Lyme disease exposure during pregnancy. This concise review describes the current state of knowledge of Lyme disease as a congenital infection and the potential effects of in utero *B. burgdorferi* exposure to the early neurodevelopment of infants and children and proposes future directions for researchers interested in developing high-quality research in this area.

## 2. Lyme Disease as a Congenital Infection

Exposure to microbes during pregnancy or delivery may affect the health of pregnant people and their fetuses. Acute Lyme disease occurs following the transmission of the spirochete *Borrelia burgdorferi* from the bite of an infected blacklegged tick. There is some scientific agreement that *B. burgdorferi* can potentially be vertically transmitted and may cause congenital infection, and it is possible that Lyme disease may have effects on the fetus, placenta, and developing child similar to other diseases caused by spirochetes, such as syphilis [9,10,11,12]. The impact of infectious exposures on a developing fetus is dependent on many factors including the gestational age at exposure, the integrity of the maternal–fetal interface, immune response and other host factors, severity of symptoms, nature of the agent, and the mechanism by which the infectious agent is transmitted to the fetus. Neurodevelopmental assessment of children exposed to congenital infection is recommended, as the effects of antenatal infectious exposures can impact developmental domains such as behavior, cognitive function, mobility, and communication. Delays in these domains may not be immediately evident in infancy, thus longitudinal follow-up to early or late childhood is required to detect them [13].

Impacts of intrauterine infectious exposure may be due to either direct exposure to the agent (i.e., *B. burgdorferi*) or to resultant changes in the placental/in utero milieu as a result of host infection or inflammatory response as has recently occurred with SARS-CoV-2 infection [14]. The mechanism(s) of possible vertical *B. burgdorferi* transmission, and physiological changes resulting from in utero exposure and/or transmission, are unclear at this time. There are three major mechanisms of vertical transmission of bacterial agents—antepartum, intrapartum, and postpartum [12]. In the case of antepartum infections, there are two pathways by which bacteria can reach the fetus including ascending infection and hematogenous infection. Bacteria that are transmitted via ascending infection will typically travel up the cervicovaginal canal, enter the uterus, and upon reaching the placenta, cause chorioamnionitis. When occurring in pregnancy, spirochetes typically will reach the placenta through hematogenous, or maternal bloodstream, infection, termed spirochetemia. This is typically seen in the most common spirochetal infection of pregnancy, caused by *Treponema pallidum* [15,16]. Intrapartum transmission of bacteria occurs when the fetus is delivered through an infected birth canal. Postpartum transmission of bacterial agents occurs following delivery and can include infection of the newborn through skin contact, aerosols, secretions, and ingestion. In the case of *B. burgdorferi*, the most likely mechanism of vertical transmission is through the hematogenous route and placental infection, as there is no evidence of the organism occurring in the cervicovaginal canal. Although there has been a single report of human milk positivity for *B. burgdorferi* [17], this mechanism of vertical infection appears extremely unlikely and it is recommended that individuals with Lyme disease continue to breastfeed their infants [18].

## 3. Placental Changes as a Possible Mechanism of Influence on Fetal and Child Development

There is almost no knowledge of the effects of *B. burgdorferi* on the placenta. Hematogenous transmission following maternal spirochetemia is the most common mechanism for maternal–fetal transmission of spirochetes. Spirochetes in maternal blood can travel throughthe uterine arterial circulation to reach the placenta via the spiral arterioles, where they can enter the intervillous space and have intimate contact with the syncytiotrophoblast, the major protective cell layer of the maternal–fetal interface. In addition, the chorionic villi possess additional defenses against infection that include fetal-derived stromal macrophages (Hofbauer cells) as well as the fetal chorionic microvasculature, which must be breached for an infectious agent to reach fetal blood. Maternal immune cells provide another source of immunological protection. However, many organisms can breach these defenses, reaching the villous circulation to infect the fetus. 

Examination of the placenta is a valuable method for the diagnosis of intrauterine transplacental transmission of a microbial agent to a fetus. Placental pathology has been instrumental in our understanding of mechanisms of vertical transmission and pathogenesis of congenital infections including Zika virus [19], SARS-CoV-2 [20], syphilis [15], mpox [21], and others. The development of antibody- and nucleic acid-based pathology techniques has been increasingly important in localizing a wide variety of infectious agents in the placenta, understanding mechanisms of transplacental transmission, and defining the immunological response to infection [22]. Even in the absence of direct transmission of a pathogen to the developing fetus, it is possible that Lyme disease and/or PTLDS may alter the placental environment as a result of infection-related immune adaptations or chronic inflammation [23,24]. Child outcomes after placental infection with *B. burgdorferi* are unclear [10,25]; thus, placental pathology holds promise for clarifying the pathogenesis of congenital *B. burgdorferi* infection.

## 4. Early Neonatal and Infant Outcomes After Lyme Disease During Pregnancy

Literature reviews assessing the impact of *B. burgdorferi* infection during pregnancy provide some evidence that gestational Lyme disease is a risk factor for adverse pregnancy outcomes, especially when untreated throughout the course of pregnancy [26,27]. Other studies report favorable outcomes at birth but do not follow infants longitudinally [10,11,25,28]. To our knowledge, none of the studies on pregnancy, fetal, or early neonatal outcomes have systematically utilized standardized measures to assess the neurodevelopmental outcomes of living children with in utero exposure to *B. burgdorferi* beyond early infancy [7].

Existing case studies and cross-sectional studies of infants exposed to Lyme disease during pregnancy also report varying effects on early child development. Clinical data from 2008 to 2020 by Trevisan et al. indicated healthy growth and weight at 1 and 2 years of age among 11 children born to women with Lyme disease in Italy; however, further neurodevelopmental assessments were not conducted [29]. An international cross-sectional study by Leavey et al. (2022) reported that rates of hypotonia, respiratory distress, and unexplained fevers were higher in the neonatal period among infants born following pregnancies with “Probable Untreated Lyme disease” compared to those with little evidence of Lyme disease infection or those who were identified as Lyme disease being both likely and treated during pregnancy [30]. Conclusions from this study echo those drawn from studies on adverse pregnancy outcomes on the importance of the treatment of acute Lyme disease during pregnancy on the fetal and infant health and development [27,30].

Among a cohort of 304 patients treated with antibiotics for an erythema migrans rash during pregnancy, Maraspin et al. (2020) reported that 15 patients’ children had developmental anomalies within the first year of life [31]. While the author noted that there may be potential explanations besides Lyme disease exposure for other unfavorable outcomes seen in the cohort such as preterm birth or fetal/perinatal death, potential explanations for the anomalies identified later in infancy were not reported or explored further [31]. Authors found no specific patterns of abnormalities reported in the literature or among their cohort, and the frequency of outcomes reported were similar to outcomes among pregnant patients without Lyme disease and are not necessarily related to a congenital infection [31]. No neurodevelopmental sequelae were reported.

## 5. Child Neurodevelopmental Outcomes After Lyme Disease During Pregnancy

It is important to note that while studies assessing pregnancy, birth, and early life outcomes are valuable for counseling families and providing recommendations for early intervention, findings cannot be extrapolated to older children. Negative findings in the neonatal or infant period are not adequate evidence to support a lack of neurodevelopmental impact later in life [27]. Similarly to the recommendations for other infections during pregnancy, it is of critical importance to follow infants exposed to Lyme disease in utero throughout childhood and the adolescent years [32,33,34]. Serial monitoring of developmental milestones and age-appropriate neurodevelopmental functions will also allow us to identify potential impacts of early intervention that may be valuable to addressing any neurodevelopmental concerns or developmental delays.

To date, no studies have conclusively found a relationship between the presence of Lyme disease during gestation and adverse neurodevelopmental outcomes in childhood or adolescence. Existing studies are limited by methodological and statistical challenges that do not allow us to fully understand the possible range of outcomes following in utero exposure to Lyme disease/PTLDS, or important variables that may influence outcomes. Thus, there remains a significant need for prospective studies using validated outcome measures to understand the impact of gestational Lyme disease exposure on the developing brain and childhood neurodevelopmental outcomes.

Research conducted in the 1990s found no influence of maternal Lyme disease infection on neurological disorders in childhood or congenital anomalies; however, it is worth noting that this research was limited by small sample sizes of patients with confirmed *B. burgdorferi* infection [28,35] and methods which relied on retrospective surveys [28,36]. Additionally, children in the abovementioned studies were not followed systematically to assess long-term development.

Nadal et al. followed infants exposed to Lyme disease in utero to age 9–17 months; however, only eleven children were clinically examined, and none underwent standardized neurodevelopmental assessments to evaluate the presence of developmental delays [37]. Bransfield et al. hypothesized that congenital Lyme disease may play a role in autism spectrum disorder in older children [38]. However, most of the supporting evidence comes from animal models of inflammation associated with maternal autoantibodies specific for *B. burgdorferi*, parent/clinician reporting of autism spectrum disorder diagnosis, and previous untreated *B. burgdorferi* infection during gestation [38,39,40]. 

A commonly cited case series by Jones et al. reported high levels of adverse neurological sequelae in children following untreated or partially treated Lyme disease during pregnancy [41]; however, this report should be interpreted with caution as the methodology is unclear and potentially biased, and other explanations for outcomes or other confounding factors are not adequately assessed [27,41]. Leavey et al.’s international cross-sectional survey found higher rates of neurological, mental health, and other issues in childhood among children whose gestational parents had “probable” and “possible” Lyme disease and were untreated during pregnancy, compared to those who were treated [30]. This result supports the hypothesis that treatment is an important factor; however, questions remain whether identified outcomes are due to congenital transmission of *B. burgdorferi*, other changes to the in utero or to the postnatal environment that are indirectly related to parental Lyme disease, or other factors. The authors also note that it is possible that children in their study had Lyme disease themselves, as opposed to or in addition to a congenital infection [30]. This too is an important confounding factor to consider, as prior studies have proposed a correlation between Lyme disease in childhood and neurodevelopmental outcomes such as mental health concerns and autism spectrum disorder [38,42,43,44,45].

## 6. Other *Borrelia* Infections in Pregnancy

Certain *Borrelia* species have been well-documented to cause poor obstetrical outcomes when infection occurs during pregnancy. These include *B. duttonii* (endemic to several countries in sub-Saharan Africa), *B. hermsii* (North America), and *B. turicatae* (North America) which leads to tick-borne relapsing fever, and *B. recurrentis*, which causes louse-borne relapsing fever [12,46,47]. *Borrelia duttonii* has been found to be associated with adverse outcomes that include spontaneous abortion, preterm delivery, low birth weight, and perinatal death [12,46,47]. Larsson et al. have devised a murine model for the study of *B. duttonii* in pregnancy that has demonstrated congenital infection in experimentally infected female mice [47]. Additional findings included fetal growth restriction, abnormalities of fetal circulation, placental damage, and decreased maternal hemoglobin levels. The placentas from experimentally infected fetal mice showed necrotic lesions associated with inflammatory cells, increased fibrin deposition, and the presence of spirochetes in the labyrinthine areas of the placenta [47]. *Borrelia* species can spread through the bloodstream following initial infection and thus have the potential to reach the placenta in infected pregnant individuals; for instance, *B. mayonii* has been identified as a highly spirochetemic organism and may therefore have higher susceptibility to vertical infection [48]. However, there are no data in the literature on vertical transmission of *B. mayonii* to date, and neurodevelopmental outcomes of children exposed to the abovementioned *Borrelia* infections in the fetal period have not yet been published.

## 7. Discussion

Scientists, doctors, and patients have been advocating high-quality research on child outcomes after parental *B. burgdorferi* infection in pregnancy for decades. Potential changes to the placental environment due to *B. burgdorferi*, including but not limited to direct parental–fetal transmission of the spirochete, may have lasting impacts on child neurodevelopment. However, research on child neurodevelopmental outcomes and factors associated with positive development after Lyme disease exposure has lagged behind other congenital infections. One conclusion that can be drawn from the literature is that treatment of acute Lyme disease during pregnancy with antibiotic medication may have a protective effect against adverse infant and child outcomes. Pregnant individuals with Lyme disease are typically treated with either oral amoxycillin or oral cefuroxime for 2–4 weeks [18,29,31,49]; doxycycline is not recommended because of its potential effects on the fetus. However, additional research is necessary to determine factors such as the duration of treatment and types of treatment for different stages of parental Lyme disease (i.e., acute Lyme disease or PTLDS) that may further protect child neurodevelopment.

Variability in the reporting of study designs and methods of existing research also complicates analysis. Different perspectives on the diagnosis of Lyme disease and varying case definitions of “Lyme disease” in the literature limit our ability to understand the mechanisms by which having Lyme disease while pregnant may influence child growth and development. The use of laboratory tests to diagnose Lyme disease also varies. For example, the review conducted by Waddell et al. in 2018 included some studies in which patients’ Lyme disease was confirmed by different types of serology, some which relied on retrospective chart review, and some who were simply considered to be “at higher risk” of Lyme disease due to living in an endemic area or reporting a history of being bitten by a tick [27]. Research on chronic symptoms after initial Lyme disease treatment (including clinician-diagnosed PTLDS) is important and must be distinguished from acute cases to better understand differences between potential impacts of *B. burgdorferi* itself on the developing fetus and impacts of pregnant patients’ immune response, inflammation, or other consequences of Lyme disease/PTLDS infection. Misclassification of cases, or failure to collect and analyze important confounding variables and differences between study participants, can have a profound impact on what conclusions can be drawn from a study. As we work to improve clinical guidance and care for families navigating the complex diagnosis of Lyme disease and/or PTLDS during pregnancy, it is our responsibility as researchers to use rigorous and ethical study methods and clear reporting to minimize study limitations and maximize the utility of our research.

Existing evidence supports the collaboration between patients, healthcare providers, payer systems, public health agencies, and researchers to move well-designed research forward to fill current knowledge gaps. As the research on Lyme disease during pregnancy and subsequent long-term child outcomes expands, it will be paramount to utilize a standard case definition of Lyme disease and/or PTLDS; ensure careful collection of confounding variables such as symptomatology, antibiotic and other treatment history, and gestational age at acute Lyme disease exposure (if applicable); and follow children into school-age and beyond using validated methods of assessing child development.

## 8. Future Directions

Because most studies to date are retrospective, cross-sectional, anecdotal, or based on case reports [10], the state of the science warrants robust longitudinal prospective cohort studies using standardized measures which follow pregnancy outcomes and infants exposed to congenital Lyme disease throughout early childhood [8]. It is not known whether experiencing PTLDS during pregnancy has a direct or indirect impact on pregnancy and infant outcomes; this is yet another important knowledge gap to be addressed. High-quality research to understand factors that influence neurodevelopment after Lyme disease and/or PTLDS during pregnancy as well as factors that impact individual child trajectories over time are of utmost importance. In order to more completely understand the pathophysiology of Lyme disease in pregnancy, there is an ongoing mixed-methods pilot study that builds upon existing research, case studies, and advocacy using a trauma-informed and patient-centered lens to assess the feasibility of studying developmental and other family impacts of Lyme disease exposure during pregnancy (Mulkey et al.; NCT06026969) [50]. In particular, it will determine the effects of in utero exposure to Lyme disease (including both acute Lyme disease and PTLDS) on pregnancy and long-term childhood neurodevelopmental outcomes. As part of this study, a perinatal pathologist will examine placentas from fetuses exposed to acute and chronic maternal *B. burgdorferi* infections using both routine and immunohistochemical studies to characterize the microscopic spectrum of abnormalities associated with this infection [50].

Early identification and referral by obstetricians, perinatologists, and other perinatal health professionals to congenital infection specialists may play a significant role in discerning and mitigating potential short-term and long-term neurodevelopmental effects of antenatal exposure to Lyme disease. Continued research and provider education on the effects of *B. burgdorferi* on the gestational environment and the developing fetal brain is essential to inform timely treatments to protect fetuses and children. Future research should also explore the potential protective effects of various types of treatment during pregnancy. Investigators interested in Lyme disease during pregnancy should prioritize the research needs identified by patients and families affected by this condition and employ robust study designs to maximize the utility of study findings.

## Data Availability

The original contributions presented in the study are included in the article. Further inquiries can be directed to the corresponding author.
